# Advances in Pediatric Fracture Diagnosis and Treatment Are Numerous but Great Challenges Remain

**DOI:** 10.3390/children9101489

**Published:** 2022-09-28

**Authors:** Christiaan J. A. van Bergen

**Affiliations:** 1 Department of Orthopaedic Surgery, Amphia Hospital, 4800 RK Breda, The Netherlands; cvanbergen@amphia.nl; Tel.: +31-76-5955000; 2 Department of Orthopaedic Surgery and Sports Medicine, Sophia Children’s Hospital, Erasmus University Medical Center, 3000 CA Rotterdam, The Netherlands

Broken bones are very common during childhood. Nevertheless, there are many uncertainties in the scientific understanding of these injuries, and the foundations of their diagnosis and treatment. The current Special Issue on pediatric fractures, therefore, aims to improve our knowledge on various specific fractures in otherwise healthy children, and offer research on general principles in certain populations such as osteogenesis imperfecta patients. The previous Editorial [1] discussed the first 14 articles published [2,3,4,5,6,7,8,9,10,11,12,13,14,15]. Subsequently, seven more high-quality original studies and one systematic review have been published in this popular Special Issue.

Two publications address pediatric facial fractures. Children’s heads are relatively heavy compared to adults and, therefore, vulnerable to injury. Tent et al. [16] studied the etiology and epidemiology of pediatric facial fractures in Romania in a 10-year retrospective study. Of 142 children and adolescents with facial fractures, the majority were diagnosed in the 13–18 age group (79%) and in boys (88%). Most were caused by interpersonal violence, followed by falls and motor vehicle accidents. The mandible was the most affected bone. This study improves our understanding on facial fracture occurrence and provides targets for prevention.

A rare facial fracture that can be seen in children is an orbital fracture. Hsieh and Liao [17] from Taiwan and China retrospectively studied the optimal timing of surgery in 23 pediatric orbital trapdoor fractures. In a comparison between early (≤3 days) and delayed (>3 days) surgery, they concluded that operation within 3 days results in a shorter recovery interval for patients with extraocular rectus muscle entrapment.

Two more upper extremity studies have been published since the first Editorial [1]. Poppelaars et al. [18], from the Netherlands, investigated the radiological elbow fat pad sign without visible fracture in children. This sign is sometimes the only abnormal finding on radiography, and may be a clue to an occult fracture. In a large international survey amongst 133 colleagues, they found that further diagnosis and treatment of this population varied widely. Hence, more uniform protocols are required. A first step is to objectify the definition of a fat pad sign. They also performed radiographic measurements and compared those with the respondents’ judgements on the presence or absence of a positive fat pad sign. A cut-off angle of 16 degrees with respect to the anterior humeral line indicated a positive anterior fat pad sign, while any visible posterior fat pad was found to be a positive sign.

One frequent cause of a fat pad sign is a radial neck fracture. Langenberg and colleagues [19] systematically reviewed the literature on pediatric radial neck fractures, particularly on the influence of fracture treatment on range of motion after at least 1 year. In total, 26 studies with a total of 551 children were included. Closed reduction without a fixation of fractures with less than 30 degrees of angulation led to an excellent range of motion. Interestingly, in fractures angulated at more than 60 degrees, Kirshner wire fixation resulted in a better range of motion than intramedullary retrograde fixation. Moreover, open reduction resulted in a loss of motion in the majority of cases.

Descending further in the human body, two studies investigated lower extremity injuries. Weel et al. [20], in a long-term retrospective study, obtained patient-reported outcomes of adolescent soccer players with avulsion fractures of the anterior inferior iliac spine at the origin of the rectus femoris. Of seven initially nonoperatively treated patients, one required surgical excision of a heterotopic ossification. Sports were resumed at 2 to 3 months after injury in all patients, and patient-reported outcomes in the long term were highly satisfactory.

Furthermore, a large Croatian study describes the results of an impressive number of 132 pediatric tibial fractures treated with elastic stable intramedullary fixation during a period of 20 years [21]. Fifteen complications occurred, of which six required reoperation due to the secondary angulation of the fracture. They concluded that elastic nailing is an effective technique with a low complication rate.

In addition, two general studies provide insight into a broad pediatric fracture population. Fuchs et al. [22] analyzed all physeal long-bone fractures registered in the United States in 2016. Almost 6% of pediatric long-bone fractures involved the physis. Out of these 3291 fractures, 59% were localized in the lower extremity, mostly the distal tibia. The majority were classified as Salter–Harris type 2. The peak age was 11 years for girls and 14 years for boys.

Finally, Traa et al. [23] addressed the physical activity of children with extremity fractures presenting at the emergency department in an observational cross-sectional study. In the children’s population (≤12 years), 56% were adequately physically active, while in the adolescent population (>12 years), only 43% were adequately physically active, with respect to the Global Recommendations on Physical Activity for Health. These data provide opportunities for injury prevention.

In conclusion, the Special Issue consists of 22 original and review articles, which together contribute to our understanding of pediatric fracture epidemiology, diagnosis, treatment and outcomes. However, many scientific questions are still unanswered and great challenges in this interesting population remain. Therefore, a second volume of this Special Issue has been launched and is open for contributions from researchers worldwide.
